# A Prospective Study on Changes in Health Status Following Flood Disaster

**DOI:** 10.4306/pi.2008.5.3.186

**Published:** 2008-09-30

**Authors:** Ji-hoon Heo, Min-Hyuk Kim, Sang-Back Koh, Samuel Noh, Joon-Ho Park, Joung-Sook Ahn, Ki-Chang Park, Jongho Shin, Seongho Min

**Affiliations:** 1 Department of Psychiatry, Yonsei University Wonju College of Medicine, Wonju, Korea.; 2 Aerospace Medical Center, Cheongju, Korea.; 3 Department of Preventive Medicine, Yonsei University Wonju College of Medicine, Wonju, Korea.; 4 Department of Psychiatry, University of Toronto, Toronto, Canada.; 5 Centre for Addiction and Mental Health, Toronto, Canada.

**Keywords:** Disaster, Flood, Depression, Post-traumatic stress disorder

## Abstract

**Objective:**

We examined changes in general health status, the prevalence of depression and post-traumatic stress disorder (PTSD) symptoms, and the existence of pre-trauma contributing factors in an agricultural population following a massive flood.

**Methods:**

Eighty-three of 160 residents of Garisan-ni, Inje-gun, Gangwon-do, were assessed using the Korean version of the 36-Item Short-Form Health Survey (SF-36-K) between April and June 2006, just prior to a massive flood. Among those initially assessed, 58 residents were available for follow-up 18 months after the flood. Participants completed the SF-36-K, Beck Depression Index (BDI), Minnesota Multiphasic Personality Inventory (MMPI)-PTSD, and the Korean version of the Impact of Event Scale-Revised (IES-R) to detect depression and PTSD. Trauma experiences were also assessed. Factors related to changes in health status were then analyzed.

**Results:**

SF-36-K total scale scores decreased significantly, suggesting a significant reduction in health-related quality of life. The largest reductions were noted in physical and social functioning. Fifty-three percent of the subjects were at least mildly depressed, and 17% had severe depression. In addition, 22% had PTSD on both the IES-R and MMPI-PTSD. Factors that contributed to the deterioration of health status following the flood were the number of disaster events and existence of depression (as assessed by the BDI).

**Conclusion:**

The flood was found to lead to deterioration of health status and to provoke depression and PTSD among the agricultural population in the mountainous region. We suggest that the number of disaster event experiences and existence of depression contriuted to changes in health status after the flood.

## Introduction

Survivors of natural disasters may suffer from both physical injuries and psychological sequelae, such as acute stress disorder, post traumatic stress disorder (PTSD), depression, and substance abuse disorders.^1^-^4^ However, research findings vary significantly according to the characteristics of the sample employed, the type of disaster, and the particular measurements employed.^5^ In Korea, most natural disasters are caused by floods and typhoons, and reliable data for flood survivors may help illuminate strategies for pre-disaster preparation and post-flood psychiatric intervention.^6^

Previous research has reported increased rates of PTSD and long-term sustainment of various psychological symptoms among flood victims.^7^ For example, survivors of the floods that occurred in Kentucky in 1981 exhibited significant increases in cognitive impairment, somatic complaints, and negative affect, as well as a significant decline in positive affect.^8^,^9^ These changes were sustained for over a year following the disaster. Furthermore, sad mood, crying spells, and feelings of hopelessness had not resolved two years after the disaster.^8^,^9^

The prevalence of PTSD among disaster victims is high. In a sample of 1,542 Hurricane Katrina victims in 2005, 19.2% were diagnosed with PTSD. The rate was higher among women than among men. Other risk factors for PTSD included non-black race, knowing someone who died in the storm, not having property insurance, enduring a longer evacuation period, and living (currently) in a newly purchased or rented house or in a temporary trailer.^10^ In Korea, the prevalence of PTSD was 41.7% among victims of Typhoon Rusa three months after the event; risk factors included disaster warning, worsening of existing disease, and prior traumatic life events.^11^ In another study, 46.6% of residents who visited emergency mobile psychiatric services 1.5 months after a massive flood in Yonchon County, Korea, were diagnosed with PTSD. Two years after the disaster, the prevalence of PTSD remained as high as 31.1%.^12^

As evidenced by these studies, the psychological impact of natural disasters is significant. However, few studies have examined changes in symptoms and disorders between pre-and post-disaster periods because natural disasters are simply difficult to anticipate. The lack of comparable pre-event data for survivors who developed PTSD restricts our ability to make inferences about the impact of the trauma.^13^,^14^ In addition, retrospective assessments of pre-trauma attributes through self-report during post-event interviews are likely to be biased by post-trauma outcomes.^15^

In 2006, we completed a brief survey in the village of Inje-gun, Gangwon-do. Two weeks after completion of the survey, a massive flood struck the hamlet on July 15^th^, 2006. In early 2008, approximately 18 months after the flood, we revisited the village to obtain data on general health status, depression, and PTSD, as well as to study potential predictors and confounders of mental health outcomes. This study reports findings from the initial and follow-up assessments of adults in the Inje community.

## Methods

### The study

Garisan-ni is a small village of about 160 people who are almost exclusively agricultural farmers. The village is a designated cohort of Gangwon province's agricultural safety management program. During the three-month period between April and June 2006, approximately half of the adult residents (83 people) participated in a survey of physical and mental health. On July 15, 2006, there was an extremely heavy downpour of rain in the mountains of Gangwon province, causing rapid flooding and large-scale landslides that seriously damaged homes and crops in the affected area, as well as roads and bridges to most of Inje-gun. Garisan-ni was one of the regions that suffered most severely from the flood. Seven of the approximately 160 residents were declared missing or dead, and most residents were evacuated to nearby schools or military camps.

Many families lived in temporary housing for up to several months, while some families moved to other regions. Within 18 months after the disaster, most houses were restored or newly built, but only some of the infrastructure, including roads and farmland, was restored.

Between January and February 2008, 18 months after the flood, we revisited 83 subjects in whom basic investigation had been conducted. A survey was administered in their home by two psychiatrists and four medical students trained for this study. A total of 67 residents (80.7%) participated in the survey. We confirmed two flood-related deaths. Other losses to follow-up included 4 moving out of the region; 2 refusing to participate; 1 having serious hearing difficulty; 1 being on long-term vacation; and 6 having been out of the region during the flood. Additionally, 9 subjects were excluded during the analysis: 6 for missing data, 2 for severe health problems before the flood, and 1 due to a pronounced rise in the 36-Item Short-Form Health Survey-Korean version (SF-36-K) score after the flood (more than 40 points). Thus, the final number of subjects included in the analysis was 58 (69.9%).

Written consents were obtained from subjects following presentation of the survey and address of the subjects' questions. The Institute Research Board of Wonju Christian Hospital at Yonsei University Wonju College of Medicine reviewed and approved the protocol and procedures.

### Measurements

#### Pre-Disaster Survey

We used the SF-36-K for the pre-disaster baseline survey and for the 18-month post-disaster follow-up survey. The self-reporting questionnaire is a standardized measure of health-related quality of life (Ware and Sherbourne et al.).^17^ The 36-item tool is a comprehensive instrument measuring physical and mental conditions in eight domains: physical functioning (PF); social functioning; role limitation due to physical conditions; role limitation due to emotional problems; mental health; vitality (VT); bodily pain (BP); and general health (GH). For all dimensions, higher scores indicate better health status.^16^,^17^ We also obtained demographic data, including the age, sex, and marital status of the respondents. Binary measures were used to proximate respondents' education (more than 9 years of schooling or completion of middle school) and income (over 10 million Korea Won or $10,000 USD). Single item questions identified status with regard to cigarette and alcohol use.

#### Post-Disaster Survey

The post-disaster follow-up survey included a short list of psychological measures. Depression was assessed using the Beck Depression Inventory (BDI). The BDI is a scale to evaluate depression symptoms based on the Diagnostic and Statistical Manual of Mental Disorders, fourth edition (DSM-IV) diagnosis of depressive disorder. The BDI is comprised of 21 items, each rated on a 4-point Likert scale of response, with higher scores indicating a higher number of depressive symptoms.^18^ Depression greater than mild severity (above 10 points) was the focus of our study. We adopted the PTSD domain of the Minnesota Multiphasic Personality Inventory (MMPIPTSD) and the Korean Version of the revised Impact of Event Scale (IES-R). The MMPI-PTSD is a 45-item scale evaluating symptoms of PTSD, each rated on binomial categories. The total score represents the seriousness of PTSD symptoms, with a score of 17 suggested as a cutoff to detect PTSD in the general population.^19^ Horowitz and colleagues developed the IES-R to measure the degree of subjective pain experienced following specific traumatic events in the last seven days. This widely used trauma experience scale is comprised of 15 questions, each being scored from 1 (no symptoms) to 4 (frequent symptoms).^20^ Higher scores on the MMPI-PTSD and IES-R indicate a higher number of PTSD symptoms or health problems.

In order to assess trauma experience, we adapted the scale used by Parslow and colleagues.^21^ The 11-item scale consists of 4 domains: uncontrollable events, including flood-related injury and damage or destruction of personal property or property owned by family/friends; controllable events, such as being personally involved in fighting the flood at home, in the neighborhood, or elsewhere; and trauma threats, such as having been put on alert or evacuated or having buildings in the suburb damaged or destroyed. An additional question focused on the subjective response to the flood experience-whether the individual felt very frightened or very upset during the flood.^21^

### Statistical analysis

All statistical analyses were performed using SAS 9.1.4. In addition to descriptive (univariate) statistics, changes in the variables measured in both the pre- and post-disaster surveys were evaluated by means of a paired t-test. Multivariate associations were assessed with binary logistic regression models for investigating the factors contributing to deterioration of health status. The logit coefficient and 95% confidential intervals were measured. Statistical probability was fixed at 5%.

## Results

### Sample

The final sample consisted of 58 adults (28 men and 30 women). As shown in Table 1, the sample was nearly evenly represented by young adults (less than 45 years of age), middle-aged adults (45-64 years), and older adults (65 years or older). The mean pre-disaster age was 53.55±14.56 years, and 86.21% of the subjects reported being currently married. The majority had no education beyond middle school (65.52%) and had an annual net income of less than $10,000 (65.52%). In addition, 87.93% of the subjects were non-smokers, while 51.72% used alcohol.

Subjects who completed the pre-disaster survey but not the follow-up survey (n=25, mean=61.20, S.D.=10.51) were older compared to those who completed both pre- and post-disaster surveys (n=58, mean=53.55, S.D.=14.56). The difference was statistically significant (t=2.37, df=81, p=0.02). There was no significant difference between the two groups in terms of sex (χ^2^=0.48, df=1, p=0.49), marital status (Fisher's exact test, p=0.13), education level (χ^2^=1.74, df=1, p=0.19), annual net income (χ^2^=0.38, df=1, p=0.54), smoking (Fisher's exact test, p=0.06), alcohol drinking (χ^2^=0.96, df=1, p=0.37), or SF-36-K scores (t=-0.82, df=22.77, p=0.42).

### Pre- and post-disaster health status

**FIGURE 1:**
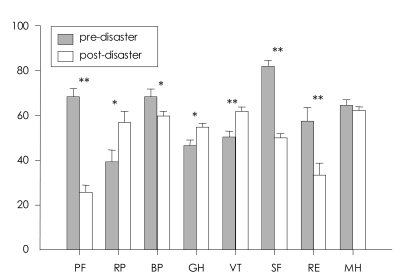
Comparison of eight categories of SF-36-K between pre- and post-disaster. ^*^p<0.05, ^**^p<0.001. SF-36-K: the Korean version of the 36-Item Short-Form Health Survey, PF: physical functioning, RP: role liitation-Physical, BP: bodily pain, GH: general health, VT: Vitality, SF: social functioning, RE: role limitation-Emotion, MH: mental health.

The results of the paired t-test for changes in pre-disaster and post-disaster SF-36-K scores are shown in Figure 1. The total SF-36-K scores decreased significantly (t=2.542, df=57, p=0.014), suggesting a significant reduction in health-related quality of life. The largest reductions were found in physical (t=9.08, p<0.001) and social functioning (t=6.86, p<0.001). More modest but statistically significant reductions were observed in role limitation due to emotional conditions (t=2.90, p=0.005) and bodily pain (t=2.03; p=0.047). The mental health domain score did not change. However, the post-disaster sample mean of self-rated general health status was significantly higher than the pre-event score (t=-2.32, p=0.024). Post-event scores of vitality and role limitation due to physical conditions (t=-3.23, p=0.002) significantly increased from the pre-event scores (t=-2.11, p=0.039).

### Risk factors

Table 1 also shows the results of the paired t-test for changes in pre- and post-disaster total SF-36-K scores by demographic characteristics. Significant reductions in the total SF-36-K scores were found among men, those of younger age (less than 45 years), those currently married, those who had an education level beyond middle school, and those with an annual income of over $10,000. Non-smokers and non-drinkers also showed a significant decline in total SF-36-K scale scores.

The pre- and post-flood changes in total SF-36-K scores were examined with the paired t-test to find associations between specific flood events and health status (Table 2). The following events were associated with a decrease in health status: 'suburb damaged or destroyed' (trauma threat), 'home or possessions damaged or destroyed', 'friend's/relative's home, possessions, or workplace damaged or destroyed', 'friend/relative died or injured due to flood', and 'owned animal that suffered due to flood' (uncontrollable traumatic events). 'Personally involved in fighting flood affecting home and neighborhood', 'did other work involving flood and its effects', 'any other controllable event' (controllable traumatic events), and 'felt very frightened or very upset' (reaction during the trauma) were also associated with a decrease in heath status. There was no decrease in health status associated with 'wasn't put on alert' or 'didn't suffer injury due to flood'.

### The health status deterioration group

Subjects who showed more than a 1-point decrease on the total post-disaster SF-36-K score were designated as the health status deterioration group (n=37, 63.79%). Binary logistic regression analysis was applied to this group to examine more specific contributing factors to the change. In Table 3, the 'number of trauma threats' indicates the number of items experienced within the domain of trauma threats, and the 'number of disaster events' indicates the number of items experienced within the domains of uncontrollable events and controllable events. The number of disaster events and a score indicating more than mild depression on the BDI were identified as contributing factors to the deterioration of health status after the flood.

### Post-disaster mental health

Nearly one-third (18 or 31.03%) of the subjects scored 24 or higher on the IES-R, which we used as a cutoff in making a clinical diagnosis of PTSD. On the MMPI-PTSD scale, 25 subjects (43.10%) scored 17 or higher, which is the suggested cutoff in identifying possible PTSD cases. Thirteen subjects (22.41%) qualified for PTSD diagnosis on both the IES-R and MMPI-PTSD.

Based on the responses to the BDI, 31 subjects (53.45%) had mild depression (BDI≥10), 11 (18.97%) had moderate depression (16≤BDI≤23), and 10 (17.24%) had severe depression (BDI≥24).

## Discussion

Our study showed a significant decrease in the post-disaster total SF-36-K score, which indicates that the flood disaster caused a deterioration in the health status of residents in the mountainous hamlet. Of the eight SF-36-K health status categories, physical functioning, role limitation due to emotional conditions, social functioning, and bodily pain were impaired or aggravated after the flood. On the contrary, general health, role limitation due to physical conditions, and vitality were improved. Lack of work due to flood-induced loss of agricultural land, or the farmer's slack season in the survey period (January to February), may explain the improvement in general health, physical limitation, and vitality. In addition, the results may be partly explained by the fact that the post-event data were collected 18 months after the flood. Over a period of 18 months following the disaster, preexisting factors (preexisting psychiatric illness, demographic risk factors, lack of supportive relationship, etc.), other adverse life events (being assaulted, etc.), and compensation and redevelopment of the hamlet may have exerted more influence over health status than the disaster itself.^22^ Indeed, a study of a large earthquake in China in 1998 found that the residents of hamlets who received large compensation for related losses had a significant difference in SCL-90 nine months after the disaster, compared with those who received relatively small compensation.^23^ We suggest that the level of compensation may be a confounding factor due to the characteristics of this sample, such as the low economic status of residents in mountainous hamlets and the inclusion of many subjects beyond 65 years of age (27.6%). An additional study is needed to determine how many survivors received compensation from the government for flood-related losses and whether such compensation affected their mental status. The lack of change in mental health status on the post-disaster SF-36-K was contrary to our initial hypothesis, which we based on previous studies suggesting that disaster aggravates mental symptoms such as anxiety and depression. However, role limitation due to emotional conditions and social functioning deteriorated, which indicates that psychosocial aspects of the residents' lives were influenced negatively by the flood experience. Finally, these findings are largely consistent with previous studies reporting increased physical pain and functional impairment following flood disasters.^10^-^12^

Residents that were younger, male, married, or had a higher educational level and income showed greater deterioration in health status following the disaster, compared to other subjects in the study. In addition, non-smokers and non-drinkers also showed greater deterioration in health status. These results are contrary to previous studies suggesting that old age, female gender, low socioeconomic status and educational level, unmarried marital status, and tobacco and alcohol use make one more vulnerable to health decreases following disaster.^10^,^21^ It is plausible that those belonging to the younger age group had higher education and income levels and were less likely to use alcohol, which enabled their awareness and reporting of subjective distress. Furthermore, male subjects may have been more influenced by the flood disaster than were female subjects due to more intimate engagement in agriculture and responsibility for family finances. However, the reason why non-smokers showed greater deterioration in health status after the flood remains unknown.

According to Parslow's research,^21^ all disaster experiences contribute to the development of PTSD: the experiences of 'being evacuated', 'friend/relative died or injured due to fires', and 'feeling very distressed during disaster' were particularly strongly associated with PTSD symptoms. In our study, most flood experiences were associated with worsening of health status, with the exception of 'being put on alert' and 'suffer(ing) injury due to flood'. If we interpret 'not being put on alert' as 'so isolated that I couldn't be alarmed, and couldn't deal with the situation', this conclusion is reasonable. Finally, subjects with more flood-related trauma events had more depressive symptoms and lower health status.

Even 18 months after the flood, 53.45% of the subjects were at least mildly depressed, and 17.24% had severe depression. In addition, 22.41% met the diagnosis for PTSD on both the IES-R and MMPI-PTSD. These rates are substantially higher than the 19.2% prevalence of PTSD found in survivors of Hurricane Katrina in 2005, six months post-event. This disparity may be attributable to differences in the age and socioeconomic status of the subjects; the mean age of the current study sample (53 years) was significantly higher than the mean age of the Katrina sample (44 years), while the educational level was significantly lower for the current study subjects.^10^,^24^

This study was meaningful because it compared health status before and after the flood based on data collected just prior to the disaster. Previous research has principally investigated psychological changes after disasters, in a retrospective manner. In addition, our study also helped illuminate factors that contributed to changes in health status after the flood.

The results of this study can be used to help direct the activities of mental health workers after disasters in agricultural areas. Through knowledge of the risk factors identified in this study, more effective public health plans may be developed by focusing limited human and financial resources on eliminating and reducing risk factors.^25^-^27^

A limitation of this study is the small number of subjects (83 subjects in the initial survey and 58 in the follow-up survey). It is possible that the subjects who died during the flood or survivors who moved to other areas suffered from more severe mental anguish; our non-examination of these individuals may have resulted in sample bias and misleading results. In addition, most subjects were middle-aged or older. Because older individuals are more vulnerable to PTSD, the results of our study, particularly with regard to the prevalence of PTSD, may be more severe^22^ than those seen in the general population. In addition, periodic follow-up is necessary to investigate changes in health status with passing time^28^ and new natural disasters, especially since seasonal floods are likely to repeat annually in Korea.

## Figures and Tables

**TABLE 1:**
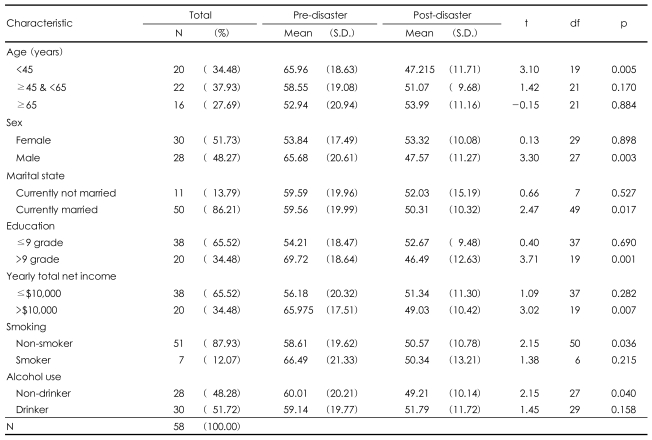
Comparison of SF-36-K scores pre- and post-disaster by demographic data

SF-36-K: the Korean version of the 36-Item Short-Form Health Survey

**TABLE 2:**
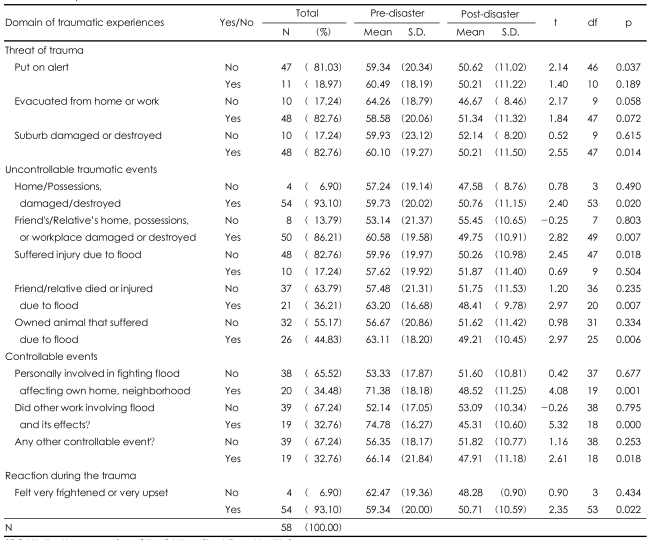
Comparison of SF-36-K scores pre- and post-disaster by traumatic experience

SF-36-K: the Korean version of the 36-Item Short-Form Health Survey

**TABLE 3:**
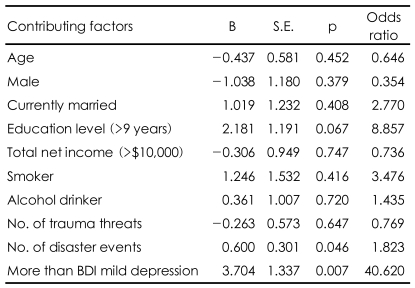
Regression analysis of factors contributing to deterioration of SF-36-K

SF-36-K: the Korean version of the 36-Item Short-Form Health Survey, BDI: Beck Depression Inventory
